# Differential Nitric Oxide Responses in Primary Cultured Keratinocytes and Fibroblasts to Visible and Near-Infrared Light

**DOI:** 10.3390/antiox13101176

**Published:** 2024-09-27

**Authors:** Augustin C. Barolet, Brice Magne, Daniel Barolet, Lucie Germain

**Affiliations:** 1 Centre de Recherche en Organogénèse Expérimentale de l’Université Laval/LOEX, Université Laval, Quebec City, QC G1V 0A6, Canada; augustin-c.barolet.1@ulaval.ca (A.C.B.); magneb@chop.edu (B.M.); 2 Regenerative Medicine Division, CHU de Quebec-Université Laval Research Centre, Quebec City, QC G1J 1Z4, Canada; 3 RoseLab Skin Optics Research Laboratory, Laval, QC H7T 0G3, Canada; daniel.barolet@mcgill.ca; 4 Dermatology Division, Department of Medicine, McGill University Health Centre, McGill University, Montreal, QC H4A 3J1, Canada; 5 Department of Surgery, Faculty of Medicine, Université Laval, Quebec City, QC G1V 0A6, Canada

**Keywords:** nitric oxide, photobiomodulation, visible light, near-infrared light, skin, 4,5-diaminofluorescein (DAF), keratinocytes, fibroblasts

## Abstract

NO is a crucial signaling molecule involved in skin health, the immune response, and the protection against environmental stressors. This study explores how different wavelengths of light, namely blue (455 nm), red (660 nm), and near infrared (NIR, 850 nm), affect nitric oxide (NO) production in skin cells. Primary keratinocytes and fibroblasts from three donors were exposed to these wavelengths, and NO production was quantified using a DAF-FM fluorescent probe. The results demonstrated that all three wavelengths stimulated NO release, with blue light showing the most pronounced effect. Specifically, blue light induced a 1.7-fold increase in NO in keratinocytes compared to red and NIR light and a 2.3-fold increase in fibroblasts compared to red light. Notably, fibroblasts exposed to NIR light produced 1.5 times more NO than those exposed to red light, while keratinocytes consistently responded more robustly across all wavelengths. In conclusion, blue light significantly boosts NO production in both keratinocytes and fibroblasts, making it the most effective wavelength. Red and NIR light, while less potent, also promote NO production and could serve as complementary therapeutic options, particularly for minimizing potential photoaging effects.

## 1. Introduction

Primary cell culture can help dissect the complex function of human skin cells, providing a simple in vitro system for studying skin physiology and pathology. These cell cultures consist mostly of primary keratinocytes and fibroblasts. These cell types have been extensively utilized to investigate various biochemical and cellular processes, including the production and release of nitric oxide (NO), which play crucial roles in skin homeostasis, the immune response, and photoprotection [1,2].

NO is a versatile signaling molecule, involved in numerous physiological processes. In the skin, it is produced by keratinocytes and fibroblasts through the action of nitric oxide synthases (NOSs), including the constitutive forms (eNOS and nNOS) and the inducible form (iNOS) [3,4]. NO can also be released through non-enzymatic pathways, such as the photolysis of nitrosothiols [5]. The detection and quantification of NO in biological systems has been greatly facilitated by the use of 4,5-diaminofluorescein (DAF), a fluorescent probe that reacts with NO to form a highly fluorescent triazole derivative [6].

The exposure of primary skin cells to different wavelengths of light, namely ultraviolet (UV), visible, and near infrared (NIR), has been shown to influence NO production through distinct mechanisms. UV light, particularly UVA (320–400 nm), penetrates the epidermis and superficial dermis [7], inducing NO release predominantly via the activation of iNOS and the photolysis of nitrosothiols. This pathway is associated with the skin’s defense mechanisms against UV-induced damage, mainly through vasodilation and ROS scavenging [8,9]. Visible light, especially in the blue spectrum (400–495 nm), also affects NO production, albeit through different molecular pathways. Blue light can penetrate the epidermis and upper dermis, affecting keratinocyte and fibroblast function by photolyzing NO derivatives, such as nitrosothiols, while also influencing NO metabolism through the activation of flavoproteins and the modulation of mitochondrial activities, leading to enhanced nitric oxide release within the skin [10]. Red light (620–700 nm) penetrates more deeply into the skin, reaching the deep dermis, where it influences mitochondrial function at this depth and enhances nitric oxide (NO) release. Near-infrared (NIR) light (700–1400 nm) penetrates even further, reaching subcutaneous adipose tissue, muscles, joints, and bones, similarly affecting mitochondrial function and NO release in these deeper tissues [11,12]. While the precise mechanisms remain unclear, it is believed that non-ionizing light interacts with and activates various cellular biomolecules, such as cytochrome c oxidase, flavoproteins, and light-sensitive ion channels, which in turn influence NO production [12].

This study aims to advance our understanding of nitric oxide (NO) release in primary human skin cells, specifically keratinocytes and fibroblasts, in response to visible and near-infrared (NIR) light. Rather than investigating the underlying molecular mechanisms or potential toxicity, this research focuses on the efficacy of light-induced NO production, which plays a critical role in cellular signaling, vasodilation, and tissue repair. By examining how different wavelengths influence NO release, this work provides key insights that can inform the refinement of photobiomodulation therapies. The use of primary human cells ensures that the findings are physiologically relevant and applicable to clinical practice. Ultimately, this research lays the groundwork for developing more targeted and safer light-based treatments in dermatology and regenerative medicine, tailored to the specific responses of skin cells to different wavelengths.

## 2. Materials and Methods

### 2.1. Primary Cell Cultures

Three primary human skin cell populations of keratinocytes and fibroblasts were used for this study. The sources of the cell populations are detailed in Table 1. Human cells were obtained from healthy skin specimens removed during surgical procedures, after obtaining informed consent (see Appendix A, Table A1 and Table A2) prior to sample collection. The study protocol was approved by the research ethics committee for the protection of human subjects (N2023-6411) at the CHU de Québec-Université Laval on 12 May 2022. For keratinocyte isolation, skin fragments were digested with 500 μg/mL of thermolysin (Sigma-Aldrich, St. Louis, MO, USA) in HEPES buffer (10 mM of 4-(2-hydroxyethyl)-1-piperazine ethane sulfonic acid, 6.7 mM of potassium chloride, 142 mM of sodium chloride, and 1 mM of calcium chloride) at 4 °C, overnight. The dermis was then separated from the epidermis using forceps. The keratinocytes were further dissociated using a trypsin/EDTA solution (0.05% trypsin [Trypsin (1:250) Gibco] and 0.01% EDTA salt [J.T. Baker, Teknisciences] prepared in PBS) at 37 °C, for 25 min. The fibroblasts were isolated from the dermis using a collagenase H solution (0.125 U/mL in Dulbecco’s modified eagle’s medium [DMEM]) at 37 °C, for 3 h, in a CO_2_ incubator. The keratinocyte and fibroblast populations were extensively characterized in terms of marker expression and morphology, as described in previous studies from our lab [13,14,15,16,17,18], which followed the same cell isolation protocol. This well-established method consistently yields predominantly pure cultures of keratinocytes or fibroblasts, with minimal contamination from other cell types.

**Figure 1:**
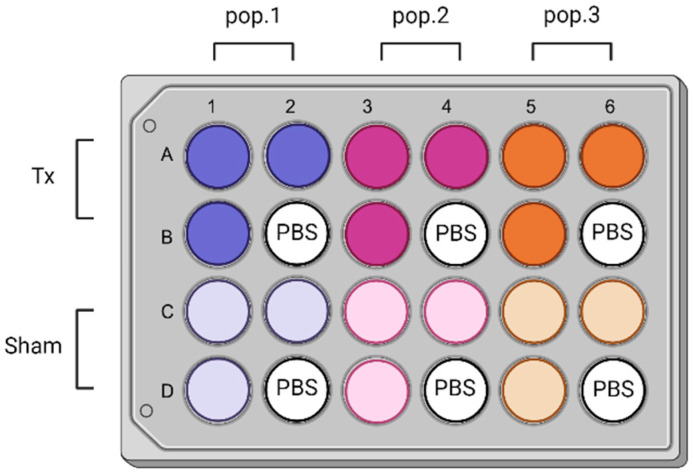
Twenty-four well plates (one per wavelength) were used to assess the effects of light exposure on different cell populations (pop. 1 (purple shades), 2 (magenta shades), and 3 (orange shades)) at an intensity of 20 mW/cm^2^ and a dosage of 15 J/cm^2^. Each column represents a specific cell population, including the treated groups (Tx) in darker shades and sham groups in lighter shades. The top two rows (**A**,**B**) are for the treated groups, while the bottom two rows (**C**,**D**) are for the sham groups. The wells labeled with “PBS” (B2, B4, B6, D2, D4, D6) serve as blank controls, which stands for phosphate-buffered saline. The blank control wells were used to account for any background signal or non-specific effects unrelated to light treatment. Aluminum tape was affixed underneath the sham wells to avoid any light exposure during the treatment. Each experiment was conducted in triplicate for each cell type (N = 3, n = 3), with a week interval between replicates. Figure 1 was sketched using BioRender (Toronto, ON, Canada).

A feeder layer of mitotically inactivated human fibroblasts (inactivated through gamma radiation at 6000 rad), with a density of 8000 cells/cm^2^, was used to amplify the primary keratinocytes. The keratinocytes were seeded on top of the feeder layer, with a density of 6500 cells/cm^2^, in a medium composed of DMEM and Ham’s F12, with a 3:1 ratio (DH). The DH medium was supplemented with 5% Fetal Clone II serum (HyClone, Wilmington, DE, USA), 5 μg/mL of insulin (Sigma-Aldrich), 0.4 μg/mL of hydrocortisone (Calbiochem, Burlington, MA, USA), 1 × 10^−6^ M isoproterenol (Sigma-Aldrich), 10 ng/mL of epidermal growth factor (EGF), and antibiotics (100 U/mL penicillin and 0.025 mg/mL gentamicin). The cell media were changed two to three times a week, until the cells reached 80% confluence. Throughout the study, the cells were maintained in an incubator at 37 °C with 5% CO_2_ to ensure proper pH regulation and prevent medium evaporation. The keratinocytes were then incubated in a DH medium, containing 5 μM DAF-FM diacetate (Invitrogen, Waltham, MA, USA) for 45 min at 37 °C, washed in PBS, and harvested with a trypsin/EDTA solution (0.05% trypsin [Trypsin (1:250) Gibco] and 0.01% EDTA salt [J.T. Baker, Teknisciences] prepared in PBS) at 37 °C for 10 min. The cells were counted using a Beckman Coulter counter, resuspended in PBS, and transferred into a 24-well plate, with a density of 800,000 cells/well, as per the plate design shown in Figure 1. They were then irradiated, and the fluorescence was immediately read using a plate reader (Varioskan LUX, ThermoFisher, Waltham, MA, USA), with 490 nm excitation, 515 nm emission, 25 flashes, and a gain setting of 70%.

The same procedure was applied to the fibroblast populations, which were grown in DMEM, supplemented with antibiotics (penicillin and gentamicin) and 10% fetal bovine serum (HyClone). Each experiment was conducted in triplicate for each cell type (N = 3, n = 3), with a week interval between experiments.

### 2.2. Configuration of the Light Source

A custom-made LED module was employed in this study. This water-cooled LED module consisted of 16 LEDs per selected wavelength, totaling 48 LEDs and irradiating in the blue (λ_peak_ = 455 nm), red (λ_peak_ = 660 nm), and NIR (λ_peak_ = 850 nm) spectra.

Each cell population received the same light treatments: 455 nm, 660 nm, and 850 nm, at 20 mW/cm^2^ and 15 J/cm^2^, for a duration of 12 min and 30 s. Prior to the experiment, the light parameters (irradiance and fluence) were validated using AlamarBlue HS (Invitrogen, USA) staining on Population 3 (K and F). This analysis showed no significant difference in the fluorescence between the irradiated samples and the sham controls across all the wavelengths (see Appendix A). A retrospective review [19] also confirmed that blue light under 30 mW/cm^2^ and 35 J/cm^2^ showed negligible differences in terms of cell viability from the controls, supporting the safety of our light parameters.

Before each treatment, the light module was calibrated with a power meter connected to a photodiode (Gentec UNO connected to silicon-type PH100-Si-HA-OD1-D0, Quebec City, QC, Canada). The module was then sanitized with isopropyl alcohol and inserted into the cell culture flow hood for the light treatment.

**Figure 2:**
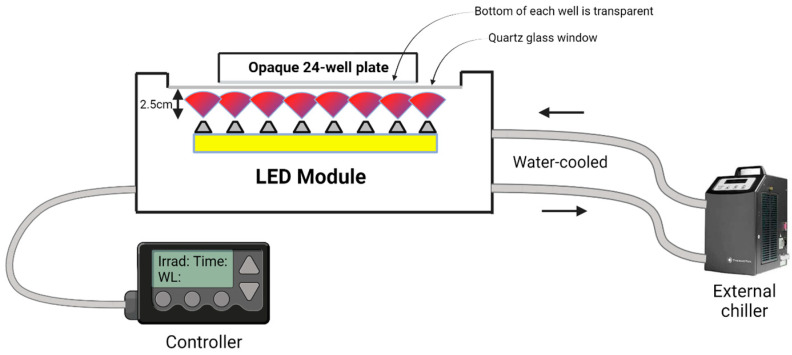
Schematic representation of the experimental setup used to irradiate cells in a 24-well plate. The setup features an opaque 24-well plate placed 2.5 cm above an LED module, which is held upside down so that the LEDs face the transparent bottom of the wells. The LED module is cooled with water to maintain a stable temperature during the irradiation process. The transparent bottom of each well allows light to penetrate and reach the cells. The setup also includes a quartz glass window that ensures uniform light distribution across the wells. Aluminum tape is applied to the transparent bottoms of certain wells to block light exposure, establishing them as sham (control) wells.

During the treatment, an opaque 24-well plate (Ibidi, Gräfelfing, Germany) was used. The plate was placed onto the light module, which was held upside down so that the LED module faced the top. The cells were positioned at a distance of 2.5 cm from the module’s LEDs (Figure 2). To prevent photon exposure to the sham wells, aluminum tape was affixed to the exterior side of the sham wells, ensuring that only the treated wells received light exposure.

### 2.3. Experimental Design

Each population was tested alongside their respective controls, which included sham light and PBS blank treatments. The experimental setup ensured that each condition was thoroughly tested, with appropriate controls to validate the results.

The data collected from the plate reader (Varioskan LUX, ThermoFisher) was then analyzed. The experimental conditions and treatments were carefully controlled and replicated to ensure the reliability and validity of the results.

### 2.4. Statistical Analysis

A linear mixed-effects model was employed to evaluate the effects of the light wavelength on the response variable, accounting for the repeated measures design of the experiment. This model was chosen to handle within-subject correlations and variability among the replicates, effectively. To test the significance of the fixed effects, we conducted post hoc pairwise comparisons with Tukey’s adjustment to control for multiple comparisons.

Residuals from the mixed-effects model were examined for normality using the Shapiro–Wilk test. For datasets exhibiting non-normal residuals, a log transformation was applied to the response variable. Specifically, in this study, a log transformation was applied to the fold changes of the keratinocyte cells to achieve normality. The R software, (version 4.4.1) along with 5 different packages [i.e., Matrix (version 1.7.0), Lme4 (version 1.1.35.5), LmerTest (version 3.1.3), Ggplot2 (version 3.5.1), Emmeans (version 1.10.3), and Dplyr (version 1.1.4)], was used to perform all the statistical analyses and create the graphs.

## 3. Results

### 3.1. Keratinocytes Release More NO under Blue Light Compared to Red or NIR Exposure

**Figure 3:**
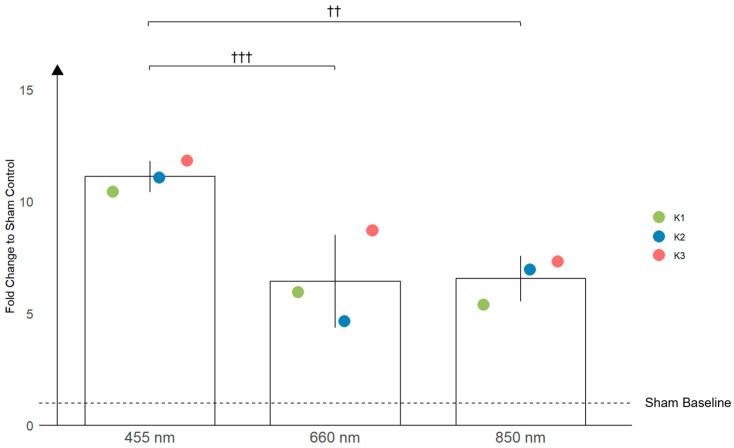
The bar graph illustrates the effects of three different wavelengths of light (455 nm, 660 nm, and 850 nm) on the production of nitric oxide (NO) in primary human keratinocytes. The data represent the mean NO production across three different donor populations (K1, K2, K3), with their respective standard deviation (SD). The fold increase in NO production is shown relative to the sham-treated controls. Statistically significant differences are indicated with crosses, with †† *p* < 0.01, and ††† *p* < 0.001. Significant differences were observed between 455 nm and 660 nm, as well as between 455 nm and 850 nm.

The results, as shown in Figure 3, depict the fold increase in NO release relative to the sham treatment for each keratinocyte population under the three different light wavelengths. Keratinocytes produced significantly more NO when exposed to 455 nm blue light compared to 660 nm red light, with a fold increase of approximately 1.7 times (*p* = 0.0007). Similarly, NO production under 455 nm blue light was 1.7 times higher than under 850 nm NIR light (*p* = 0.0019). These findings indicate a strong wavelength-dependent response in terms of NO production, with blue light consistently resulting in the highest NO release across all the keratinocyte populations (Figure 3). Further analysis comparing the treatment to the sham control revealed highly significant increases in NO production for all the wavelengths, with all the conditions being statistically different from the respective sham controls (*p* < 0.0001).

### 3.2. Blue Light Elicits Stronger NO Production in Fibroblasts Compared to Red and NIR Light, with Unexpectedly Higher Levels at the 850 nm than the 660 nm Wavelength

**Figure 4:**
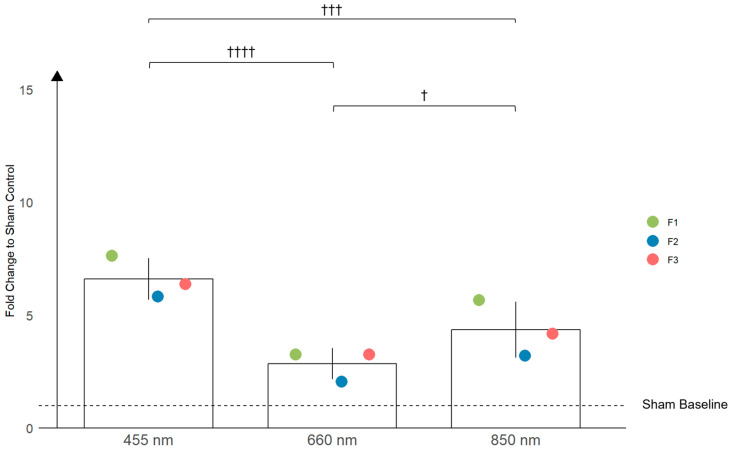
The bar graph illustrates the effects of three different wavelengths of light (455 nm, 660 nm, and 850 nm) on the production of nitric oxide (NO) in primary fibroblasts from three different patients: F1, F2, and F3. The fold increase in NO production is shown relative to the sham-treated controls. Each bar represents the mean ± standard deviation (SD) across the different populations. Statistically significant differences between the different light treatments are indicated with crosses †, with † *p* < 0.05, ††† *p* < 0.001, and †††† *p* < 0.0001.

The mixed-effects model analysis revealed significant differences in NO production among the fibroblast populations when exposed to different light wavelengths (Figure 4). Specifically, fibroblasts produced approximately 2.3 times more NO when treated with 455 nm blue light compared to 660 nm red light (*p* < 0.0001), and 1.5 times more NO when compared to 850 nm NIR light (*p* < 0.001). Surprisingly, NO production was approximately 1.5 times higher under 850 nm NIR light compared to 660 nm red light (*p* < 0.05). Further analysis showed that NO production under each wavelength (455 nm, 660 nm, and 850 nm) was statistically different from the respective sham controls, with all comparisons yielding *p*-values of less than 0.0001. These findings highlight the significant impact of light treatments on NO production in fibroblasts.

### 3.3. Keratinocytes Exhibit Stronger NO Production Response to Blue Light Compared to Fibroblasts across Different Wavelengths

The comparison of NO production between keratinocytes and fibroblasts across different wavelengths reveals distinct differences in the amplitude of the response. For keratinocytes, the highest mean NO production was observed at 455 nm (11.12), with a notable decrease at 660 nm (6.45) and 850 nm (6.57) wavelengths of light. This suggests that keratinocytes are particularly responsive to blue light, producing significantly more NO compared to red and NIR wavelengths.

In contrast, fibroblasts exhibited a more modest overall response. The highest NO production in fibroblasts was also observed at 455 nm, but the amplitude of the response was lower compared to keratinocytes (6.62 vs. 11.12). The NO production at 660 nm and 850 nm wavelengths of light in fibroblasts was considerably lower than in keratinocytes (2.87 vs. 6.45 and 4.36 vs. 6.57, respectively), indicating that fibroblasts are less sensitive to these wavelengths.

## 4. Discussion

Our study shows how different wavelengths of light (455 nm, 660 nm, and 850 nm) affect NO production in primary human keratinocytes and fibroblasts derived from three different donors. The key findings in this study indicate that (i) all tested light treatments induce an increased NO production in both primary human fibroblasts and keratinocytes, (ii) blue light is the treatment modality that promotes the strongest NO release in both cell types, and (iii) keratinocytes produce more NO than fibroblasts when exposed to any tested light treatment.

### 4.1. Consistent Blue Light-Induced NO Production in Keratinocytes with Notable Variability among the Populations

Blue light consistently induced the highest NO release across all the keratinocyte populations. These results reinforce the well-established role of blue light in promoting NO production in the skin, as documented in previous research [10,19,20,21].

However, the data also reveal intriguing differences in the response to light exposure among the three keratinocyte populations. Notably, Population K3 exhibited slightly different behavior, particularly under 660 nm irradiation, where the NO release was notably different compared to K1 and K2. This marked difference at 660 nm could be related to underlying genetic factors or variations in skin phototype, which influence how keratinocytes respond to light. Additionally, the age of the donor may also play an important role in this variability, potentially affecting the efficiency of NO production pathways, such as the activation of nitric oxide synthases (NOSs) (e.g., inducible NOS) [10,19]. These factors suggest that keratinocytes from individuals with different skin phototypes, or with different ages, exhibit unique adaptive responses to light, resulting in the differential behavior seen in this study.

Despite these variations, the overall trend remains clear: 455 nm blue light is the most effective wavelength for inducing NO production across all the keratinocyte populations. The lesser, yet still significant, responses observed under 660 nm and 850 nm wavelengths further support the unique efficacy of blue light in stimulating these cells. The consistent superiority of blue light suggests that it interacts more efficiently with specific chromophores in the skin, such as opsins, flavins, and porphyrins, which are known to absorb blue light more effectively than red or NIR light [11].

Additionally, the data indicate a higher variability in NO production among the keratinocyte populations compared to fibroblasts. The standard deviations of all the values taken from the replicates show greater variability in keratinocytes, although these specific SDs are not shown in Figure 3 and Figure 4. Instead, the figures display the standard deviations from the overall mean per wavelength, which show similar variability between keratinocytes and fibroblasts. This suggests that while individual responses within keratinocyte populations are more variable, the average response per wavelength does not differ significantly in terms of their variability between the two cell types. This finding underscores the complexity of keratinocyte responses, which may be influenced by age-related and UV-induced factors [22,23]. For example, keratinocytes from younger individuals tend to respond more robustly to UV light, possibly due to a higher proportion of proliferative cells compared to adults, who have more differentiated cells [24]. These factors could influence the expression and activity of NO-producing enzymes, further contributing to the observed variability in keratinocyte responses. This highlights the need for careful interpretation when assessing NO production across different wavelengths and cell types.

### 4.2. Blue Light Elicits Stronger NO Production in Fibroblasts Compared to Red and NIR Light, with Unexpectedly Higher Levels under the 850 nm than the 660 nm Wavelength

The analysis of NO production in fibroblasts under different light wavelengths reveals a consistent trend across all the fibroblast populations (F1, F2, F3). Blue light at 455 nm consistently induced the highest NO production, surpassing both 660 nm red light and 850 nm NIR light.

Interestingly, 850 nm NIR light also stimulated significantly more NO production than 660 nm red light, with approximately 1.5 times higher NO levels observed under NIR exposure (*p* < 0.05). This finding was consistent across all the fibroblast populations.

These results suggest that while blue light remains the most effective wavelength for inducing NO production in fibroblasts, NIR light at 850 nm also plays a significant role, outperforming red light at 660 nm. Given that NIR light penetrates deeper into the skin in vivo, it is possible that fibroblasts, which reside in the deeper dermis, may have evolved to be more responsive to NIR exposure. However, this is speculative and based on the known properties of NIR light penetration in human tissue [25]. Further studies using in vivo models are necessary to confirm this hypothesis.

Furthermore, the superior effect of 850 nm light compared to 660 nm light in releasing NO may be due to thermal effects, as NIR light is better absorbed by water molecules, leading to a localized increase in temperature. This could enhance enzymatic activity, including that of NOSs, resulting in higher NO production [26,27,28]. Interestingly, even though both fibroblasts and keratinocytes were irradiated in the same medium (PBS) under identical experimental conditions, fibroblasts exhibited significantly greater NO release under NIR light compared to red light, while the keratinocytes did not. This difference could be attributed to the intrinsic characteristics of the cell types, such as the higher intracellular water content in fibroblasts [29], making them more sensitive to the thermal effects of NIR light. Furthermore, fibroblasts and keratinocytes may have different expressions of NOS or heat-sensitive enzymes, influencing their response to light [30]. These findings highlight that, even under controlled conditions, the distinct biology of these cell types may drive the differential responses to light irradiation.

We also observed that all the fibroblast populations exhibited remarkably consistent responses to the different light treatments, with minimal variability in NO production, as indicated by the smaller standard deviations across the populations (see Appendix A). This lower variability in NO production among the fibroblasts, compared to the keratinocytes, indicates a more stable and homogenous response across these cell types, reinforcing the reliability of these wavelengths, especially 455 nm, in consistently stimulating NO release.

### 4.3. Impact of Blue Light-Induced ROS on NO Signaling and DAF Fluorescence

DAF-FM was selected due to its high specificity and sensitivity for detecting intracellular NO in living cells. DAF-FM has been widely used in similar studies because it provides a robust and reliable measure of NO production in real-time [31,32,33], allowing the dynamic changes in NO levels to be monitored following light exposure.

One important consideration in this study is the potential for increased DAF (4,5-diaminofluorescein) fluorescence due to oxidative reactions induced by blue light exposure. Blue light is known to generate higher levels of reactive oxygen species (ROS) because of its high energy and interaction with cellular chromophores, such as flavins and porphyrins, which are abundant in skin cells [34]. These ROS can react with the DAF probe, leading to increased fluorescence signals [35], potentially affecting the sensitivity and accuracy of the NO measurements.

While this concern is valid, it is important to note that ROS not only interact with the DAF probe, but they may also play a role in the NO release itself. The interaction between ROS and NO pathways is complex and could contribute to the observed increase in NO production under blue light [36,37,38]. Future studies should aim to quantify ROS levels, alongside NO, to better understand their relationship and the specific contributions of ROS to the observed effects.

### 4.4. Implications for Light-Based Therapies

The findings from this study strongly support the use of blue light (455 nm) as the most effective wavelength for increasing nitric oxide (NO) production in both keratinocytes and fibroblasts. Given the consistent and superior response observed across all the cell populations, blue light emerges as the optimal choice for light-based therapies aimed at enhancing NO release in the skin.

However, it is important to acknowledge that blue light, while highly effective, is more ionizing and can potentially contribute to premature photoaging, partially due to its ability to generate reactive oxygen species (ROS) [39,40] and persistent hyperpigmentation via opsin 3 receptors, which are primarily responsible for the modulation of melanocyte differentiation and pigment production [41]. This highlights a potential trade-off in regard to therapeutic applications: while blue light maximizes NO release, it may also increase the risk of photoaging and hyperpigmentation over prolonged or repeated treatments [34,42].

In this context, treatment with 660 nm red light and 850 nm near-infrared (NIR) light may serve as alternative or complementary options. Although these wavelengths induce lower NO production compared to blue light, they still facilitate significant NO release, while potentially mitigating the adverse effects associated with blue light. The deeper penetration of NIR light, coupled with its lower ionizing potential, may provide added benefits, especially in targeting fibroblasts within the dermis, while minimizing the risk of surface-level photoaging and hyperpigmentation.

## 5. Conclusions

In conclusion, this study demonstrates that all three wavelengths, blue light (455 nm), red light (660 nm), and near-infrared light (850 nm), induced significant nitric oxide (NO) release in both primary human keratinocytes and fibroblasts, with blue light being the most effective among them. Keratinocytes exhibited a more robust level of NO release across all three wavelengths compared to fibroblasts, which, although responsive, generally produced lower fold increases. Despite the effectiveness of blue light in maximizing NO release, its potential contribution to photoaging and hyperpigmentation suggests that 660 nm red light and 850 nm NIR light could be considered as alternative therapeutic options. Although these wavelengths are less potent, they still promote significant NO release. Further research will be essential to refine these light-based therapies and optimize their safety and efficacy.

## Figures and Tables

**Table 1:** Donor information on skin cell populations.

ID	Cell Type	Source	Donor′s Sex	Donor′s Age	Fitzpatrick Index
Population 1	K * and F **	Breast Surgery	Female	55 years	II
Population 2	K and F	Facelift	Female	60 years	II
Population 3	K and F	Foreskin	Male	<1 month	IV

* Keratinocytes and ** Fibroblasts.

## Data Availability

The data supporting the findings of this study are available from the corresponding author upon request.

## Appendix A

**Table A1:** Viability analysis on Population 3 (fibroblasts (F) and keratinocytes (K)).

	Fibroblasts			Keratinocytes		
Irradiance *, Fluence *	455 nm	660 nm	850 nm	455 nm	660 nm	850 nm
5 mW, 10J	1.04	1.04	1.03	1.02	0.98	0.99
10 mW, 10J	1.01	1.02	1.03	0.99	1.03	1.02
15 mW, 10J	0.97	1.12	1.07	1.03	1.02	1.03
20 mW, 10J	0.99	1.09	1.09	1.02	1.06	1.05
5 mW, 15J	1.01	1.11	0.99	1.02	1.01	1.05
10 mW, 15J	0.97	1.17	1.03	0.99	1.06	1.02
15 mW, 15J	0.99	1.01	1.05	1.03	1.14	1.01
20 mW, 15J	0.96	1.04	1.01	0.98	1.12	1.03

* All light parameters (Irradiance & Fluence) are given per cm^2^, with mW/cm^2^ representing the irradiance and J/cm^2^ representing the fluence.

### The Viability Analysis Method

The keratinocytes were seeded at a density of 6500 cells/cm^2^ on top of an inactive feeder layer of human fibroblasts (8000 cells/cm^2^), while the fibroblasts were seeded directly at 8000 cells/cm^2^ in a 12-well plate. Two days after seeding, the cells were irradiated, with the bottom of the sham control wells covered in aluminum foil to prevent light exposure. Forty-eight hours post-irradiation, the cells were washed twice with PBS and incubated for 3 h in fresh cell media supplemented with AlamarBlue HS (Invitrogen, USA), at a 1:10 ratio. Following the incubation, 100 μL of the media from each well was transferred to a 96-well plate, and the fluorescence was measured using a plate reader (Varioskan LUX, ThermoFisher, USA), with an excitation wavelength of 560 nm and an emission wavelength of 590 nm, using 15 flashes, and a 40% gain setting.

**Table A2:** Mean and standard deviation (SD) for the individual cell populations of fibroblasts and keratinocytes.

Wavelength	Cell Population	Mean	Standard Deviation
455 nm	F1	7.638007	2.948261
455 nm	F2	5.83564	1.310756
455 nm	F3	6.346638	0.409673
660 nm	F1	3.264572	1.226903
660 nm	F2	2.065564	0.343041
660 nm	F3	3.19994	0.7239476
850 nm	F1	5.682373	0.4192602
850 nm	F2	3.209916	0.2970698
850 nm	F3	4.224489	0.2612656
455 nm	K1	10.440256	2.1218991
455 nm	K2	11.078691	0.6286809
455 nm	K3	11.83777	4.8197893
660 nm	K1	5.95976	1.6264189
660 nm	K2	4.66611	0.820669
660 nm	K3	8.723922	4.4463689
850 nm	K1	5.401495	1.840381
850 nm	K2	6.975029	0.5356716
850 nm	K3	7.32995	2.5079849

## References

[1] Ponec M., Weerheim A., Kempenaar J., Mulder A., Gooris G.S., Bouwstra J., Mommaas A.M. (1997). The formation of competent barrier lipids in reconstructed human epidermis requires the presence of vitamin C. J. Investig. Dermatol..

[2] Boelsma E., Verhoeven M.C., Ponec M. (1999). Reconstruction of a human skin equivalent using a spontaneously transformed keratinocyte cell line (HaCaT). J. Investig. Dermatol..

[3] Förstermann U., Sessa W.C. (2012). Nitric oxide synthases: Regulation and function. Eur. Heart J..

[4] Bogdan C. (2001). Nitric oxide and the immune response. Nat. Immunol..

[5] Suschek C.V., Oplander C., van Faassen E.E. (2010). Non-enzymatic NO production in human skin: Effect of UVA on cutaneous NO stores. Nitric Oxide.

[6] Kojima H., Sakurai K., Kikuchi K., Kawahara S., Kirino Y., Nagoshi H., Hirata Y., Nagano T. (1998). Development of a fluorescent indicator for nitric oxide based on the fluorescein chromophore. Chem. Pharm. Bull..

[7] Meinhardt M., Krebs R., Anders A., Heinrich U., Tronnier H. (2009). Absorption Spectra of Human Skin In Vivo in the Ultraviolet Wavelength Range Measured by Optoacoustics. Photochem. Photobiol..

[8] Mowbray M., McLintock S., Weerakoon R., Lomatschinsky N., Jones S., Rossi A.G., Weller R.B. (2009). Enzyme-independent NO stores in human skin: Quantification and influence of UV radiation. J. Investig. Dermatol..

[9] Paunel A.N., Dejam A., Thelen S., Kirsch M., Horstjann M., Gharini P., Mürtz M., Kelm M., de Groot H., Kolb-Bachofen V. (2005). Enzyme-independent nitric oxide formation during UVA challenge of human skin: Characterization, molecular sources, and mechanisms. Free Radic. Biol. Med..

[10] Opländer C., Deck A., Volkmar C.M., Kirsch M., Liebmann J., Born M., van Abeelen F., van Faassen E.E., Kröncke K.-D., Windolf J. (2013). Mechanism and biological relevance of blue-light (420–453 nm)-induced nonenzymatic nitric oxide generation from photolabile nitric oxide derivates in human skin in vitro and in vivo. Free Radic. Biol. Med..

[11] Hamblin M.R. (2018). Mechanisms and Mitochondrial Redox Signaling in Photobiomodulation. Photochem. Photobiol..

[12] Karu T.I., Pyatibrat L.V., Afanasyeva N.I. (2005). Cellular effects of low power laser therapy can be mediated by nitric oxide. Lasers Surg. Med..

[13] Goyer B., Pereira U., Magne B., Larouche D., Kearns-Turcotte S., Rochette P.J., Martin L., Germain L. (2019). Impact of ultraviolet radiation on dermal and epidermal DNA damage in a human pigmented bilayered skin substitute. J. Tissue Eng. Regen. Med..

[14] Fradette J., Larouche D., Fugère C., Guignard R., Beauparlant A., Couture V., Caouette-Laberge L., Roy A., Germain L. (2003). Normal human Merkel cells are present in epidermal cell populations isolated and cultured from glabrous and hairy skin sites. J. Investig. Dermatol..

[15] Attiogbe E., Larochelle S., Chaib Y., Mainzer C., Mauroux A., Bordes S., Closs B., Gilbert C., Moulin V.J. (2023). An in vitro autologous, vascularized, and immunocompetent Tissue Engineered Skin model obtained by the self-assembled approach. Acta Biomater..

[16] Germain L., Rouabhia M., Guignard R., Carrier L., Bouvard V., Auger F.A. (1993). Improvement of human keratinocyte isolation and culture using thermolysin. Burns.

[17] Michel M., Török N., Godbout M.J., Lussier M., Gaudreau P., Royal A., Germain L. (1996). Keratin 19 as a biochemical marker of skin stem cells in vivo and in vitro: Keratin 19 expressing cells are differentially localized in function of anatomic sites, and their number varies with donor age and culture stage. J. Cell Sci..

[18] Laplante A.F., Germain L., Auger F.A., Moulin V. (2001). Mechanisms of wound reepithelialization: Hints from a tissue-engineered reconstructed skin to long-standing questions. FASEB J..

[19] Uzunbajakava N.E., Tobin D.J., Botchkareva N.V., Dierickx C., Bjerring P., Town G. (2022). Highlighting nuances of blue light phototherapy: Mechanisms and safety considerations. J. Biophotonics.

[20] Stern M., Broja M., Sansone R., Grone M., Skene S.S., Liebmann J., Suschek C.V., Born M., Kelm M., Heiss C. (2018). Blue light exposure decreases systolic blood pressure, arterial stiffness, and improves endothelial function in humans. Eur. J. Prev. Cardiol..

[21] Garza Z.C.F., Born M., Hilbers P.A.J., van Riel N.A.W., Liebmann J. (2018). Visible Blue Light Therapy: Molecular Mechanisms and Therapeutic Opportunities. Curr. Med. Chem..

[22] Tanveer M.A., Rashid H., Tasduq S.A. (2023). Molecular basis of skin photoaging and therapeutic interventions by plant-derived natural product ingredients: A comprehensive review. Heliyon.

[23] Debacq-Chainiaux F., Leduc C., Verbeke A., Toussaint O. (2012). UV, stress and aging. Dermato-Endocrinology.

[24] Bertrand-Vallery V., Boilan E., Ninane N., Demazy C., Friguet B., Toussaint O., Poumay Y., Debacq-Chainiaux F. (2010). Repeated exposures to UVB induce differentiation rather than senescence of human keratinocytes lacking p16INK-4A. Biogerontology.

[25] Mignon C., Uzunbajakava N.E., Castellano-Pellicena I., Botchkareva N.V., Tobin D.J. (2018). Differential response of human dermal fibroblast subpopulations to visible and near-infrared light: Potential of photobiomodulation for addressing cutaneous conditions. Lasers Surg. Med..

[26] Suschek C.V., Wondrak G.T. (2016). Nitric Oxide Derivatives and Skin Environmental Exposure to Light: From Molecular Pathways to Therapeutic Opportunities. Skin Stress Response Pathways: Environmental Factors and Molecular Opportunities.

[27] Kroncke K.D., Suschek C.V., Kolb-Bachofen V. (2000). Implications of inducible nitric oxide synthase expression and enzyme activity. Antioxid. Redox Signal..

[28] Eells J.T., Henry M.M., Summerfelt P., Wong-Riley M.T., Buchmann E.V., Kane M., Whelan N.T., Whelan H.T. (2003). Therapeutic photobiomodulation for methanol-induced retinal toxicity. Proc. Natl. Acad. Sci. USA.

[29] Levin J., Maibach H. (2008). Human skin buffering capacity: An overview. Ski. Res. Technol..

[30] Schäfer M., Werner S. (2007). Transcriptional Control of Wound Repair. Annu. Rev. Cell Dev. Biol..

[31] Kojima H., Nakatsubo N., Kikuchi K., Kawahara S., Kirino Y., Nagoshi H., Hirata Y., Nagano T. (1998). Detection and imaging of nitric oxide with novel fluorescent indicators: Diaminofluoresceins. Anal. Chem..

[32] Ridnour L.A., Thomas D.D., Mancardi D., Espey M.G., Miranda K.M., Paolocci N., Feelisch M., Fukuto J., Wink D.A. (2004). The chemistry of nitrosative stress induced by nitric oxide and reactive nitrogen oxide species. Putting perspective on stressful biological situations. Biol. Chem..

[33] Pope N.J., Powell S.M., Wigle J.C., Denton M.L. (2020). Wavelength- and irradiance-dependent changes in intracellular nitric oxide level. J. Biomed. Opt..

[34] Oplander C., Hidding S., Werners F.B., Born M., Pallua N., Suschek C.V. (2011). Effects of blue light irradiation on human dermal fibroblasts. J. Photochem. Photobiol. B.

[35] Ruemer S., Krischke M., Fekete A., Lesch M., Mueller M.J., Kaiser W.M. (2016). Methods to Detect Nitric Oxide in Plants: Are DAFs Really Measuring NO?. Methods Mol. Biol..

[36] Radi R. (2013). Peroxynitrite, a stealthy biological oxidant. J. Biol. Chem..

[37] Thomas D.D., Ridnour L.A., Isenberg J.S., Flores-Santana W., Switzer C.H., Donzelli S., Hussain P., Vecoli C., Paolocci N., Ambs S. (2008). The chemical biology of nitric oxide: Implications in cellular signaling. Free Radic. Biol. Med..

[38] Weller R. (2003). Nitric oxide: A key mediator in cutaneous physiology. Clin. Exp. Dermatol..

[39] Godley B.F., Shamsi F.A., Liang F.Q., Jarrett S.G., Davies S., Boulton M. (2005). Blue light induces mitochondrial DNA damage and free radical production in epithelial cells. J. Biol. Chem..

[40] Coats J.G., Maktabi B., Abou-Dahech M.S., Baki G. (2021). Blue Light Protection, Part I—Effects of blue light on the skin. J. Cosmet. Dermatol..

[41] Regazzetti C., Sormani L., Debayle D., Bernerd F., Tulic M.K., De Donatis G.M., Chignon-Sicard B., Rocchi S., Passeron T. (2018). Melanocytes Sense Blue Light and Regulate Pigmentation through Opsin-3. J. Investig. Dermatol..

[42] Arjmandi N., Mortazavi G., Zarei S., Faraz M., Mortazavi S.A.R. (2018). Can Light Emitted from Smartphone Screens and Taking Selfies Cause Premature Aging and Wrinkles?. J. Biomed. Phys. Eng..

